# Diabetes-preventive molecular mechanisms of breast versus formula feeding: new insights into the impact of milk on stem cell Wnt signaling

**DOI:** 10.3389/fnut.2025.1652297

**Published:** 2025-07-29

**Authors:** Bodo C. Melnik, Ralf Weiskirchen, Sabine Weiskirchen, Wolfgang Stremmel, Swen M. John, Claus Leitzmann, Gerd Schmitz

**Affiliations:** ^1^Department of Dermatology, Environmental Medicine and Health Theory, University of Osnabrück, Osnabrück, Germany; ^2^Institute of Molecular Pathobiochemistry, Experimental Gene Therapy and Clinical Chemistry (IFMPEGKC), RWTH University Hospital Aachen, Aachen, Germany; ^3^Praxis for Internal Medicine, Baden-Baden, Germany; ^4^Institute for Interdisciplinary Dermatological Prevention and Rehabilitation (iDerm), University of Osnabrück, Osnabrück, Germany; ^5^Institut für Ernährungswissenschaft, University of Gießen, Gießen, Germany; ^6^Institute of Clinical Chemistry and Laboratory Medicine, University Hospital of Regensburg, Regensburg, Germany

**Keywords:** breastfeeding, diabetes mellitus, milk exosome, microRNA, formula feeding, wingless signaling, β-cell, diabetes prevention

## Abstract

Human milk serves as a transmitter for epigenetic programming involved in postnatal tissue development and organ maturation of the infant. In contrast to formula feeding (FF), prolonged breastfeeding (BF) has been associated with diabetes-preventive effects. Polymorphisms of the transcription factor 7-like 2 (TCF7L2), the key downstream effector of Wingless (Wnt) signaling, increase the risk of diabetes mellitus. Wnt signaling is crucial for β-cell development and proliferation. However, there is limited information regarding Wnt/β-catenin/TCF7L2-dependent effects of BF versus FF on postnatal β-cell progenitor cell development, β-cell proliferation and β-cell mass expansion. The objective of our literature review is to collect and analyze data to provide translational evidence that different components of human milk promote Wnt signaling. We will specifically focus on the variations in Wnt signaling in enteroendocrine L-cells and pancreatic β-cells in response to either FF or BF. FF-induced overstimulation of mTORC1 may suppress Wnt gene expression through S6K1-mediated histone H3K27 trimethylation (H3K27me3). Moreover, the absence of milk exosomal miRNAs in formula that target mRNAs of crucial Wnt inhibitors, as well as reduced levels of eicosapentaenoic acid and glutamine in formula, may further hinder appropriate Wnt signaling, negatively impacting intestinal stem cells, enteroendocrine L-cells and potentially β-cell progenitor cells. Overall, the evidence presented supports the conclusion that FF has a detrimental impact on the Wnt/β-catenin/TCF7L2-regulated enteroendocrine-islet axis, disrupting proper β-cell maturation and proliferation. We propose that human milk, compared to formula, offers optimized conditions for physiological Wnt signaling promoting adequate neonatal β-cell mass expansion, which could explain the early diabetes-preventive effects of prolonged BF.

## 1 Introduction

A systematic review confirmed the association of prolonged breastfeeding (BF) with a reduced risk of type 2 diabetes (T2DM) (1). In a population with a high prevalence of T2DM, the Pima Indians, infants who were exclusively breastfed had significantly lower rates of T2DM than those who were exclusively bottlefed (2). Similarly, a case control study reported that BF reduces the risk of T2DM among native Canadian children (3). According to a systematic review and meta-analysis of 11 high-quality studies, the odds ratio for T2DM was lower among subjects who had been breastfed [pooled odds ratio: 0.65 (95% CI: 0.49; 0.86)] (4), recently confirmed by an updated meta-analysis by Horta et al. (5). Over the last two decades, several reviews (6–8) including recommendations of the Lancet Series Breastfeeding Group (9), the American Diabetes Association (10), and the Word Health Organization (11) have promoted prolonged BF for the prevention of T2DM later in an infant’s life. However, the question of how and why BF prevents T2DM is still uncertain and a matter of speculation, as T2DM is still regarded as a non-communicable disease (12).

**Figure 1:**
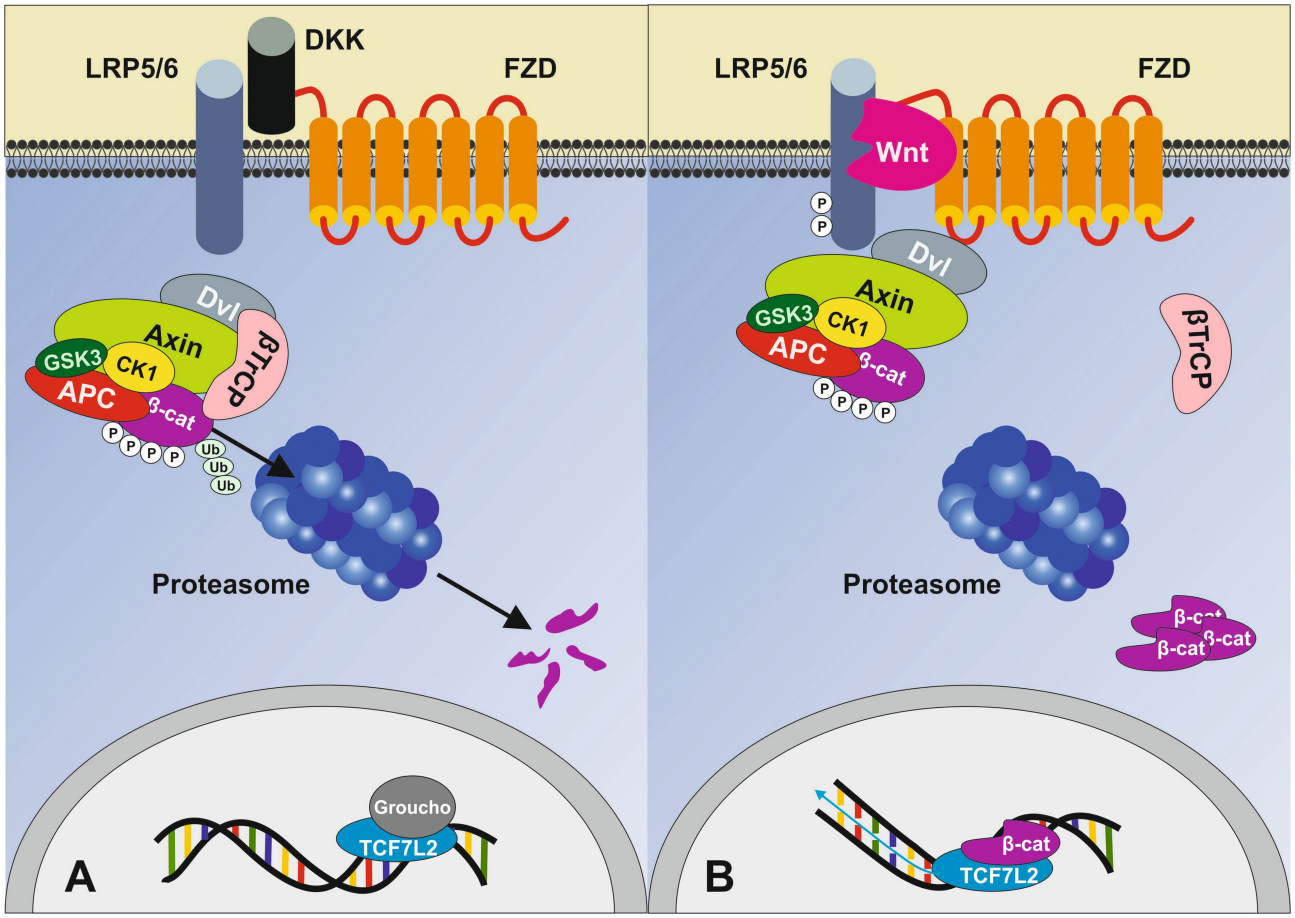
Simplified overview of the canonical Wnt signaling pathway. **(A)** In the absence of Wnt ligands, cytoplasmic β-catenin is phosphorylated by the destruction complex (DC), which includes Axin, adenomatosis polyposis coli (APC), glycogen synthase kinase 3 (GSK3) and casein kinase 1 (CK1). Phosphorylation of β-catenin within this complex by CK1 and GSK3 targets it for ubiquitination (Ub) and subsequent proteolytic destruction. Without nuclear β-catenin, TCF7L2 engages with Groucho (TLE3 in β-cells), preventing the transcription of Wnt target genes. **(B)** When Wnt protein binds, it leads to the heterodimerization of the Frizzled receptor (FZD) with low-density lipoprotein receptor-related protein 5/6 (LRP5/6) followed by conformational changes resulting in the phosphorylation of LRP5/6 intracellular domain recruiting Axin and the DC to the cell membrane thereby inhibiting its activity. Subsequently, stable, non-phosphorylated β-catenin accumulates in the cytoplasm and translocates into the nucleus. The β-catenin/TCF7L2 complex regulates the expression of various Wnt target genes. Dickkopf (DKK) is a specific Wnt inhibitor that antagonizes Wnt signaling through direct interaction with the LRP5/6 receptor. Adapted from Napolitano et al. (92), licensed under CC BY 4.0.

The transcription factor 7-like 2 (*TCF7L2*) gene is a downstream effector of the canonical Wnt/*β*-catenin signaling pathway and represents the most potent locus known for T2DM risk (13–17), linking the pathogenesis of T2DM to altered Wnt/β-catenin/TCF7L2 signaling. Figure 1 represents the canonical Wnt signaling pathway that culminates in the nuclear action of the master transcription factor TCF7L2.

Takamoto et al. (18) generated genetically engineered mice (DN mice), in which the expression of the dominant-negative form of TCF7L2 was driven under a rat insulin promoter. A marked reduction in the β-cell area and whole-pancreas insulin content was observed in both newborn and adult DN mice. Similarly, Shu et al. (19) used pancreatic sections from three mouse models (high-fat diet, exendin-4 and streptozotocin-treated mice) as well as from healthy individuals and patients with T2DM to investigate the association of β-cell regeneration in relation to TCF7L2 levels. In human isolated exocrine tissue, TCF7L2 overexpression induced proliferation of pancreatic duct cells and small islet-like cell cluster formation next to duct cells. Liu et al. (20) reported that glucagon-like peptide 1 (GLP-1) and its agonist, exendin-4 (Exd4), induce Wnt signaling in pancreatic β-cells, both isolated islets, and in INS-1 cells. Basal and GLP-1 agonist-induced proliferation of β-cells requires active Wnt signaling. Cyclin D1 and c-Myc, determinants of cell proliferation, are up-regulated by Exd4. Inhibition of Wnt signaling by small interfering RNAs to β-catenin or a dominant-negative TCF7L2 decreases both basal and Exd4-induced β-cell proliferation (20). Le Bacquer et al. (21) demonstrated that the most common diabetes-associated TCF7L2 variant rs7903146 risk allele is associated with reduced total islet numbers and impaired insulin secretion.

TCF7L2 acts as a master regulator of pancreatic β-cells, orchestrating the expression of key genes crucial for β-cell proliferation, survival and mass expansion (20–26), incretin responsiveness (26, 27), inulin production (28), and insulin secretion (29). Importantly the interplay between TCF7L2 and the expression of GLP-1 receptor (GLP1R) and glucose-dependent insulinotropic polypeptide (GIP) receptor (GIPR) regulates β-cell survival and function (26). Reduced TCF7L2 protein levels in T2DM are associated with decreased GIP- and GLP-1 receptor expression and impaired β-cell function. The TCF7L2 variant rs7903146 seems to impact the risk of T2DM by reducing the sensitivity of β-cells to incretins (30). Rulifson et al. (31) provided direct evidence that Wnt signaling controls pancreatic β-cell proliferation. Adding purified Wnt3a protein to cultured β-cells or islets increased the expression of paired-like homeodomain transcription factor 2 (PITX2) and cyclin D, crucial regulators of β-cell cycle progression, leading to enhanced β-cell proliferation *in vitro*. Activated β-catenin expression in pancreatic β-cells induced β-cell expansion, increased insulin production and serum levels, and improved glucose handling. Conversely, Axin expression, a potent negative regulator of Wnt signaling, in β-cells resulted in reduced PITX2 and cyclin D2 expression, decreased neonatal β-cell expansion and mass and impaired glucose tolerance. Gui et al. (32) confirmed that Wnt3a regulates the proliferation of the clonal β-cell line NIT-1, with these effects being completely inhibited by the Wnt signaling inhibitor Dickkopf 1 (DKK1). In accordance, Figeac et al. (33) demonstrated that Wnt/β-catenin signaling regulates neonatal growth and regeneration of β-cells in normal and diabetic rats. Patients with *APC* mutations and activated Wnt signaling show increased expression of c-Myc (34). Hou et al. (35) showed that TCF7L2 activates the promoter of c-Myc, a key transcription factor that promotes cell proliferation. Boutant et al. (36) showed that the chicken ovalbumin upstream promoter transcription factor 2 (COUP-TF2; encoded by *NR2F2*) is required for GLP-1 activation of the β-catenin-dependent pathway and its expression is under the control of TCF7L2. Notably, COUP-TF2 increases β-cell numbers during the neonatal period in human islets and rat β-cells (36).

**Figure 2:**
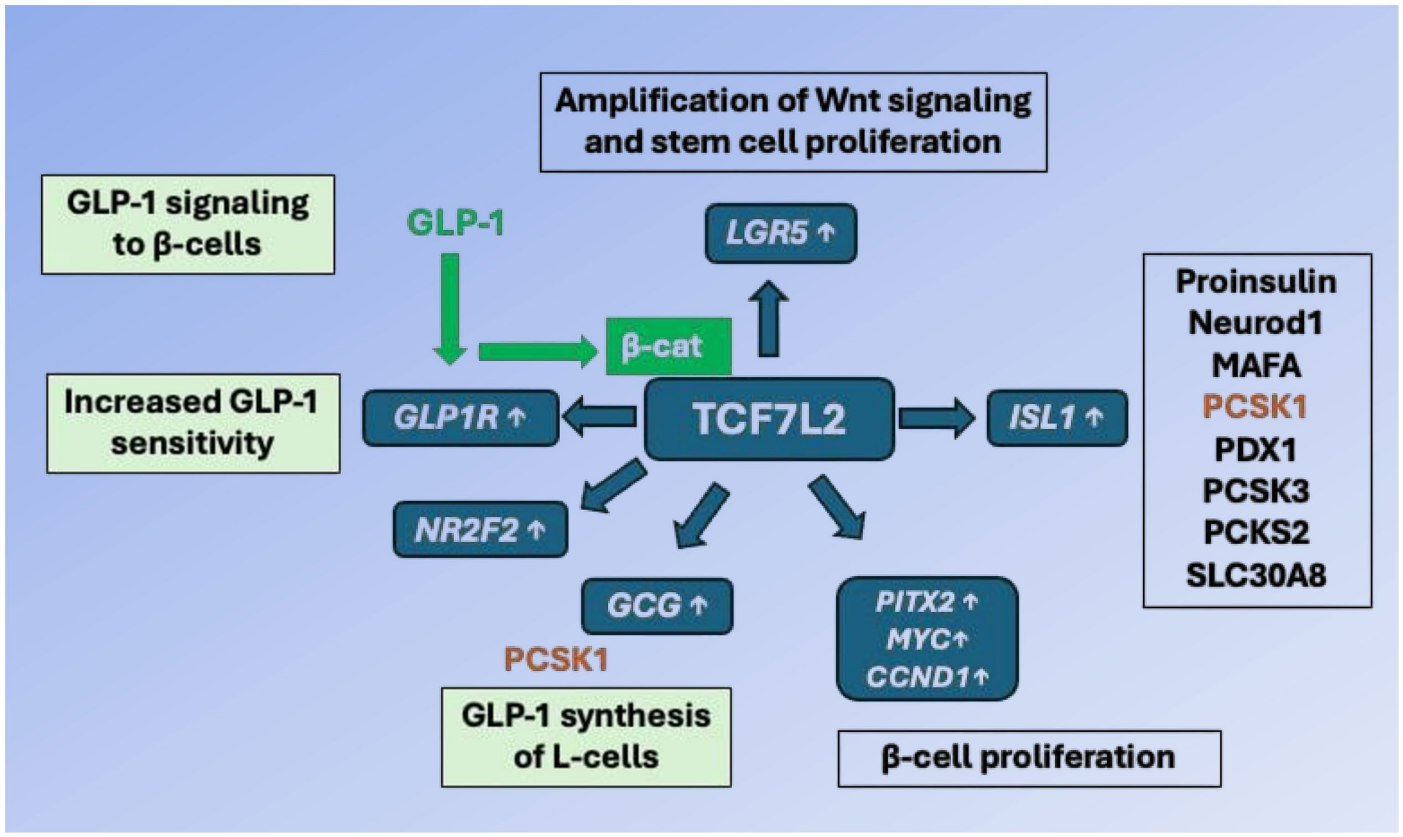
TCF7L2-dependent gene regulation of the entero-insular axis. In enteroendocrine L-cells, TCF7L2 upregulates the expression of the proglucagon gene (GCG). After proteolytic cleavage of proglucagon by proprotein convertase, subtilisin/kexin-type 1 (PCSK1), glucagon-like peptide 1 (GLP-1) is generated. GLP-1 binds to GLP-1 receptor (GLP1R) of pancreatic β-cells and via cAMP/protein kinase A activation stabilizes β-catenin (β-cat). The activated β-catenin/TCF7L2 complex promotes the expression of critical target genes including *MYC*, *PITX2* (β-cell proliferation), *ISL1* (β-cell identity and function), *GCG* (GLP-1 generation), *LGR5* (stem cell marker, augmentation of Wnt signaling), *GLP1R* (β-cell responsiveness to GLP-1) and *NR2F2* (activation of GLP-1 signaling). TCF7L2 thus integrates and maintains the molecular cross talk between enteroendocrine L-cells and pancreatic β-cells. For further explanation of gene symbols, see Glossary.

Taken together, substantial evidence underlines the importance of Wnt signaling for β-cell proliferation, mass expansion and insulin secretion (37–40). Figure 2 illustrates the importance of TCF7L2-controlled gene expression in GLP-1 producing enteroendocrine L-cells and GLP-1 responding pancreatic β-cells.

Yoshihara et al. (41) generated human islet-like organoids from induced pluripotent stem cells and demonstrated that non-canonical Wnt4 signaling drives the metabolic maturation necessary for robust *ex vivo* glucose-stimulated insulin secretion (GSIS). Recently, Katsumoto et al. (42) showed that Wnt4 is activated in β-cells as they mature, suggesting that during the early postnatal period, Wnt4 is inconsistently activated in β-cells and signals for maturation.

To understand the molecular mechanisms of the diabetes-preventive effects of physiological BF compared to artificial FF during the neonatal period, we searched for metabolic, endocrine, and epigenetic data and pathways affecting Wnt/β-catenin/TCF7L2 signaling, as well as differences between both feeding/programming types (BF versus FF).

## 2 Wnt/β-catenin signals are involved in alveologenesis, lactation, and exosome exocytosis

Wnt signaling plays a fundamental role in mammary gland development and alveologenesis (43, 44). A differential regulation of the Wnt gene family has been observed during pregnancy and lactation (45). Morales et al. (46) showed that lactation requires the expression of Na/H exchanger regulatory factor 1/ERM-binding phosphoprotein 50 (NHERF1/EBP50). The prolactin receptor (PRLR)-STAT5 signaling serves as the central axis triggering the differentiation of secretory mammary alveolar cells. In successfully lactating glands, NHERF1 is massively upregulated and forms complexes with PRLR, β-catenin, E-cadherin, and ezrin at the alveolar basal membrane, establishing basal polarity. In NHERF1-deficient glands, basal polarity is disrupted, PRLR levels and basal membrane localization are lost, and downstream STAT5 activation decreases, leading to a reduction in milk protein synthesis (46). Intruguingly, Zhang et al. (47) recently demonstated in bovine mammary gland epithelial cells (MAC-T cells) that calgranulin B (S100A9) expression upregulates milk protein synthesis, i.e., the expressions of caseins CSN1S1, CSN2, and CSN3 via upregulation of Wnt- and PI3K-mTORC1 signaling.

During lactation and milk secretion, human mammary epithelial cells release a large number of milk exosomes daily, estimated to be around 2.2 × 10^11^ exosomes/mL (48). It is not yet known if Wnt signaling plays a role in the regulation of milk exosome production and exocytosis. However, the activation of the Wnt/β-catenin signaling pathway has been shown to enhance exosome production in human umbilical cord mesenchymal stem cells (hucMSCs) (49).

## 3 Disturbed EZH2/Wnt signaling by formula feeding

**Figure 3:**
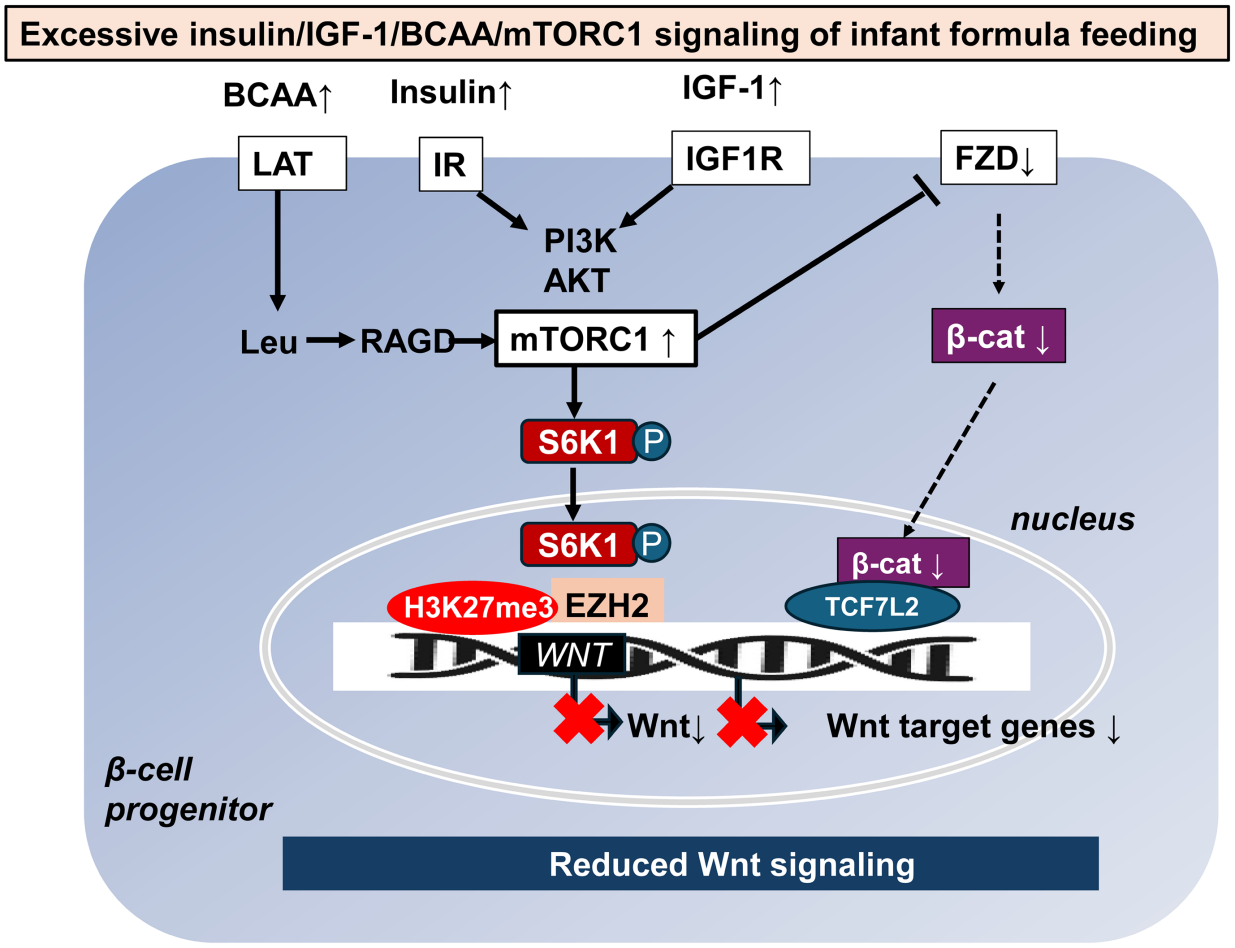
Potential mTORC1-mediated mechanisms of reduced Wnt signaling caused by formula feeding. Increased formula feeding leads to elevated levels of branched-chain amino acids (BCAAs), insulin, and insulin-like growth factor 1 (IGF-1), which in turn over-activate mechanistic target of rapamycin complex 1 (mTORC1) and its downstream kinase S6K1. Once phosphorylated S6K1 enters the nucleus, triggers enhancer of zeste homolog 2 (EZH2) to trimethylate histone 3 at lysine 27, creating a negative regulatory complex that suppresses *WNT* gene expression. In addition, overactivation of mTORC1 suppresses the availability of frizzled (FZD) receptors on the cell membrane thereby reducing Wnt signaling and Wnt target gene expression. β-cat: β-catenin, LAT: L-type amino acid transporter, Leu: leucine, RAGD: Ras-related GTP binding protein D, IR: insulin receptor, IGF1R: insulin-like growth factor 1 receptor, PI3K: phosphatidylinositol 3-kinase, and Akt: Akt kinase (protein kinase B).

FF, especially feeding of formula with high protein content, has been shown to increase serum levels of insulin, insulin-like growth factor 1 (IGF-1) and essential branched-chain amino acids (BCAAs) in human (50) and Rhesus infants (51–53) compared to natural BF. Insulin/IGF-1 and BCAAs are critical signals that activate the growth factor- and amino acid-sensitive kinase mechanistic target of rapamycin complex 1 (mTORC1) and its downstream product, the kinase S6K1 (54–57). Protein-rich FF appears to overstimulate mTORC1/S6K1 signaling (58–60). Studies have shown that mesenchymal stem cells (MSCs) prior to becoming adipogenic, mTORC1-activated (phosphorylated) S6K1 enters the nucleus (61, 62), recruits enhancer of zeste homolog 2 (EZH2) to histone 3 (H3), mediating H3K27 trimethylation (H3K27me3) at Wnt gene loci (62), ultimately resulting in Wnt target gene suppression that facilitates adipogenesis (Figure 3) (63).

In addition, Zeng et al. (64) reported that mTORC1 signaling regulates the cell surface level of the Wnt receptor Frizzled (FZD) in a Dishevelled (DVL)-dependent manner by influencing the association of DVL and the clathrin AP-2 adaptor. Notably, sustained mTORC1 activation impairs Wnt/β-catenin signaling, leading to the loss of stemness in intestinal organoids ex vivo and primitive intestinal progenitors *in vivo* (64).

Wang et al. (65) demonstrated that Wnt signaling is activated during adipose-derived stem cell (ADSC) differentiation into islet β-cells. When induced with Wnt3a, ADSCs expressed markers of β-cells, such as PDX1, CK19, nestin, insulin, and C-peptide proteins, indicating successful differentiation. The expression of TCF7L2 and β-catenin mRNA as well as β-catenin protein levels increased. Shi et al. (66) investigated the role of Wnt signaling during human ADSC differentiation into insulin-producing cells (IPCs) and concluded that Wnt/β-catenin signaling may be involved in maturation, but not differentiation of IPCs.

Xu et al. (67) reported that deletion of EZH2 at the pancreatic progenitor stage enhanced the production of endocrine progenitors and β-cells. Inhibition of EZH2 in embryonic pancreas explants and in human embryonic stem cell (ESC) cultures increased endocrine progenitors *in vitro*. According to Fontcuberta-PiSunyer et al. (68) inhibition of EZH2 enhances both the transactivation ability of Neurogenin3 in cultured cells and the formation of insulin-producing cells during directed differentiation from pluripotent cells. In addition, EZH2 is able to trimethylate β-catenin at lysine 49 (β-catMe3), which acts as a transcriptional co-repressor (69).

Recently, Al-Hasani et al. (70) showed that transient stimulation of exocrine cells derived from juvenile and adult type 1 diabetes mellitus (T1DM) donors with the EZH2 inhibitors GSK126 and Tazemetostat influences a phenotypic shift towards a *β*-like cell identity. The transition from repressed to permissive chromatin states is dependent on bivalent H3K27me3 and H3K4me3 chromatin modifications. Targeting EZH2 may thus be fundamental to β-cell regenerative potential (70). Therefore, excessive stimulation of endocrine growth factor signaling (insulin, IGF-1, BCAAs) by formulas may disrupt the proper epigenetic regulation of EZH2-controlled Wnt signaling, negatively impacting the population of β-cell progenitor cells.

## 4 LGR5: stem cell marker and amplifier of Wnt signaling in β-cell progenitors

**Figure 4:**
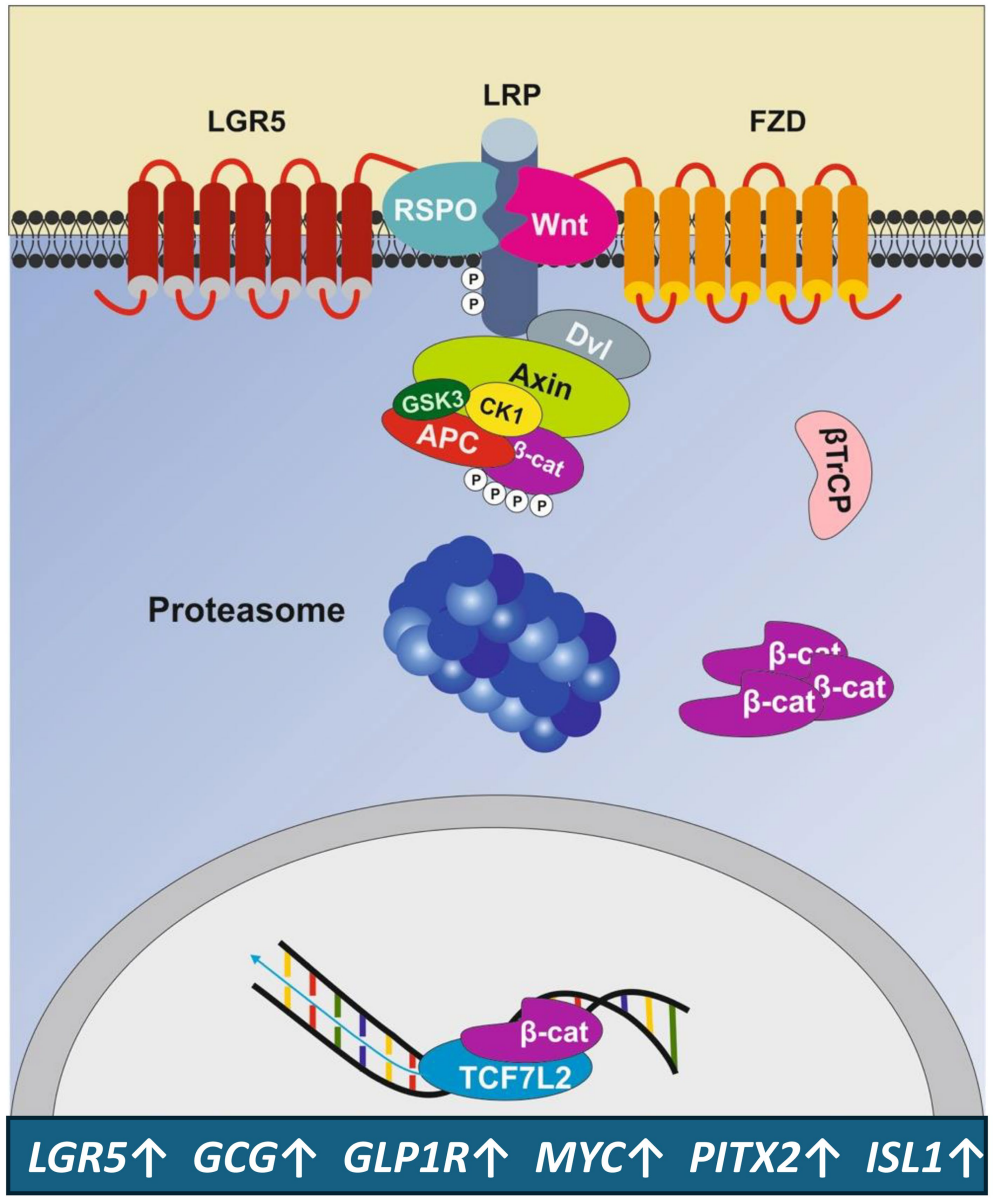
Amplification of Wnt signaling by the LGR5 receptor. The Wnt target gene and stem cell marker LGR5 enhances Wnt signaling, leading to the upregulation of genes that promote the expansion of LGR5^+^ β-cell progenitors and β-cell proliferation. For gene symbols, see Glossary.

Importantly, the stem-cell marker leucine-rich repeat-containing G-protein-coupled receptor 5 (LGR5) is expressed in pancreatic islets and co-localizes with Nanog and insulin in clusters of β-cells (71). LGR5 is a cognate receptor for R-spondins (RSPO). After binding of its RSPO ligand, LGR5 forms a potentiating complex with the Wnt protein-bound frizzled receptor (FZD)/lipoprotein-related receptor 5/6 (LRP5/6) augmenting downstream Wnt signaling and enhancing Wnt target gene expression (72–76). Notably, LGR5 itself has been identified as a Wnt target gene (77), which strengthes Wnt signaling (78). The LGR5/R-spondin complex acts by neutralizing ring finger protein 43 (RNF43) and zinc finger and ring finger protein 3 (ZNRF3), two transmembrane E3 ligases that remove Wnt receptors from the stem cell surface (78–80) The related RNF43 and ZNRF3 transmembrane E3 ubiquitin ligases are uniquely expressed in LGR5^+^ stem cells (80). Figure 4 shows key target genes upregulated by Wnt/LGR5 signaling.

Recently, Rodriguez et al. (81) provided evidence for the existence of a population of cells within and in the proximity of the ducts that transiently express LGR5 during late gestational stages in mice. LGR5 has been identified in stem cells of actively self-renewing tissues including the liver, intestine, skin and hair follicles (82–85). Remarkably, the conversion of *α*-cells to β-cells in the postpartum mouse pancreas involved LGR5^+^ progeny (81). A spontaneous lineage conversion of α-cells to β-cells occurred specifically after parturition and has been suggested to represent a novel mechanism counterbalancing against β-cell mass reduction (81). Remarkably, Gouw et al. (86) used healthy canine pancreatic ductal organoids to show that LGR5 and SOX9-expressing pancreatic ductal cells could differentiate into β-cells expressing key β-cell-related genes including pancreatic and duodenal homeobox 1 (PDX1), NK6 homeobox 1 (NKX6.1), glucose transporter type 2 (GLUT2), proprotein convertase subtilisin/kexin type 1 (PCSK1), and low levels of insulin. Importantly, Andersson-Rolf et al. (87) identified the Wnt target gene LGR5 as a marker for a tripotent stem/progenitor cell of the human fetal pancreas. Organoids derived from single LGR5^+^ cells are capable of long-term expansion *in vitro* and generation of the three main epithelial cell lineages that make up the mammalian pancreas. LGR5 expression follows the temporal expression of PDX1 suggesting a sequential activation during progenitor cell differentiation.

Accumulated evidence supports the view that Wnt signaling promotes β-cell proliferation and mass expansion (18–25, 31–33), critically involving the promotion of LRG5^+^ progenitor cells (71, 81), that exhibit the capacity for potentiated Wnt signaling. It is important to note that glucagon-like peptide-1 (GLP-1), which induces β-cell proliferation, requires active Wnt signaling (20). Inhibition of Wnt signaling by small interfering RNAs to β-catenin or a dominant-negative TCF7L2 decreases both basal and Exd4-induced β-cell proliferation (20). In accordance, geniposide increased β-cell proliferation in diabetic mice and triggered small islet-like cell cluster formation as a result of β-cell neogenesis from ductal epithelium, which was well correlated with an increase in TCF7L2 expression (88). Overstimulation of mTORC1/S6K1/EZH2/H3K27me3 signaling, induced by protein-rich FF, may impair Wnt signaling, which impairs LRG5^+^ β-cell progenitor development but promotes adipocyte stem cell (ACS) expansion and obesity risk (89).

Among the multiple molecular regulators, transcription factors, incretins and growth factors are key promoters of postnatal β-cell mass expansion (90, 91). Remarkably, Wnt signaling, which upregulates LRG5, c-MYC and PITX2, plays a fundamental role operating at early steps of stem cell/progenitor cell development critical for β-cell proliferation and mass expansion (22, 24, 28, 92).

The Wnt-β-catenin signaling pathway is an evolutionarily conserved cell–cell communication system that is important for stem cell renewal, cell proliferation and cell differentiation during embryogenesis, lineage specification, and adult tissue homeostasis (93, 94). It is conceivable that BF, a sophisticated system for maternal-neonatal programming, represents an evolutionarily optimized signaling platform that effectively maintains appropriate Wnt signaling levels for regular postnatal Wnt-dependent β-cell development and β-cell mass expansion. TCF7L2 polymorphisms with reduced functional activity increase the risk of T2DM (16, 19, 20). Experimental TCF7L2 deletions reduce β-cell mass (20), while TCF7L2 overexpression promotes β-cell proliferation (86). These findings clearly highlight the importance of Wnt signaling for β-cell development and homeostasis.

## 5 Human milk and human milk exosomes activate intestinal Wnt and GLP-1 signaling

The expression of the stem cell gene LGR5 has been identified at the bottoms of crypts in the small intestine and colon as well as in hair follicles, where LGR5 marks cycling cells with stem cell properties (84, 95–97). Notably, cycling LGR5^+^ stem cells facilitate very long-term self-renewal of small intestine, colon, and hair follicles (98). Dong et al. (99) recently demonstrated that human milk derived exosomes (HMDEs) enhanced intestinal stem cell (ISC) proliferation, as shown by a significant increase in the relative gene expression of LGR5 following HMDE administration (6.33 ± 3.01, *p* < 0.05). This effect was not observed in cells treated with HMDE-free milk (2.07 ± 0.99), compared to the control (1.00 ± 0.85). Furthermore, mRNA expression of Axin2, c-Myc, and cyclin D1 genes of the Wnt/β-catenin axis in ISCs treated with HMDEs (6.99 ± 2.34, 4.21 ± 1.68, 6.17 ± 2.22, respectively, *p* < 0.05 for all), were significantly upregulated compared to the control. In the presence of the Wnt/β-catenin signaling inhibitor carnosic acid, cell viability was significantly decreased (98). Maghraby et al. (100) demonstrated that bovine milk EVs, including exosomes, activate the Wnt pathway in the murine intestine, as evidenced by increased β-catenin staining and ISC proliferation associated with increased LGR5 expression. Hu et al. (101) recently confirmed in a murine experimental model of necrotizing enterocolitis that the addition of HBME restored epithelial regeneration, as evidenced by the number of ileum crypts and increased LGR5 expression in ISCs.

Zeve et al. (102) demonstrated the differentiation of human enteroendocrine cells (EECs) derived from ISCs. Therefore, HMDE may not only promote ISC proliferation but also the differentiation of GLP-1-producing enteroendocrine L cells, indicating a milk-gut-β-cell signaling axis. Intriguingly, Smith et al. (103) reported that human milk supplementation, as opposed to formula, improved the growth and differentiation of intestinal organoids. This resulted in larger organoids during the growth phase and organoids with longer and wider buds during differentiation compared to formula. Ki67 staining confirmed the proliferative nature of milk-supplemented organoids, while chromogranin A staining showed that human milk-supplemented organoids induced the highest EEC differentiation. Human milk supplementation upregulated genes involved in the Wnt signaling pathway, supporting early and robust EEC differentiation. Additionally, human milk-supplemented organoids downregulated negative Wnt regulators such as NOTUM (NOTUM, palmitoleoyl-protein carboxylesterase), NKD inhibitor of Wnt signaling pathway 1 (NKD1), and ZNRF3, leading to the induction of Wnt signaling and cell-cycle, consistent with the increase in Ki67 staining observed in milk-exposed organoids. Conversely, formula supplementation resulted in decreased expression of *CTNNB1*, the gene encoding β-catenin, and downstream effectors including TCF3 and TCF7.

The proglucagon gene (*GCG*) encodes the incretin hormone GLP-1, which is produced in the intestinal endocrine L cells. Importantly, TCF7L2, the downstream effector of Wnt signaling, controls the transcription of *GCG* in endocrine L-cells, which produce the incretin GLP-1 (104–106). Liu and Habener (20) provided evidence that Wnt signaling mediates GLP-1-induced β-cell proliferation. GLP-1/GLP1R-activated cAMP/PKA signaling stabilizes β-catenin and thus Wnt signaling, a key pathway in the maintenance and differentiation of β-cell progenitors. Inhibition of Wnt signaling by small interfering RNAs to β-catenin or a dominant-negative TCF7L2 decreases both basal and Exd4-induced β-cell proliferation (19).

Therefore, breastmilk and HMDEs, in contrast to HMDE-free formula, exert superior effects on ISC maturation, including the Wnt-dependent GLP-1 production that enhances GLP1-Wnt-regulated β-cell proliferation. β-catenin/TCF7L2 not only controls the production of GLP-1 but also the function of GLP-1 (27). In contrast to formula, GLP-1 is a bioactive component of human milk (107).

Remarkably, the common anti-diabetic drug metformin increased GLP-1 secretion in L-cells and db/db mice, stimulating the nuclear translocation of β-catenin and TOPflash reporter activity. However, gene depletion of β-catenin or enhancement of mutation of the TCF7L2 binding site offset the action of GLP-1 (108). Bahne et al. (109) demonstrated that metformin has a direct and AMP-activated protein kinase (AMPK)-dependent effect on GLP-1-secreting L cells, leading to an increase in postprandial GLP-1 secretion. Notably, AMPK phosphorylates β-catenin at Ser552, enhancing its stability and Wnt signaling (110). Kang et al. (111) recently showed that the GLP-1 agonist Exd-4 improves tau hyperphosphorylation and cognitive impairment in T2DM by acting on the Wnt/β-catenin/NeuroD1 pathway. Thus, the most common antidiabetic drugs promote Wnt signaling.

## 6 Potential direct interaction of breastmilk exosomes with islet β-cell progenitors

There is a recent interest in inter-organ crosstalk to understand the pathogenesis of T2DM (112) highlighting the significant role of extracellular vesicles (EVs), especially exosomes in mediating inter-organ communication in T2DM (113, 114). β-cells maintain crosstalk with various exosomes derived from adipose tissue (115, 116), muscle (117), placenta (118, 119) and liver (120). It has recently been proposed that during lactation HBMEs and their miRNAs maintain maternal-neonatal communication, reaching the systemic circulation (121–128) and may target the infant’s pancreatic islets β-cells (129, 130). Not only do human and bovine exosomes increase Wnt signaling and Wnt-dependent LGR5 expression in ISCs (99–101), but also bovine colostrum-derived exosomes, also accelerate the hair cycle transition from telogen to anagen phase by activating the Wnt/β-catenin pathway (131). Thus, milk exosomes appear to possess an intrinsic capacity for activating Wnt signaling preferentially activating recipient stem cells.

**Figure 5:**
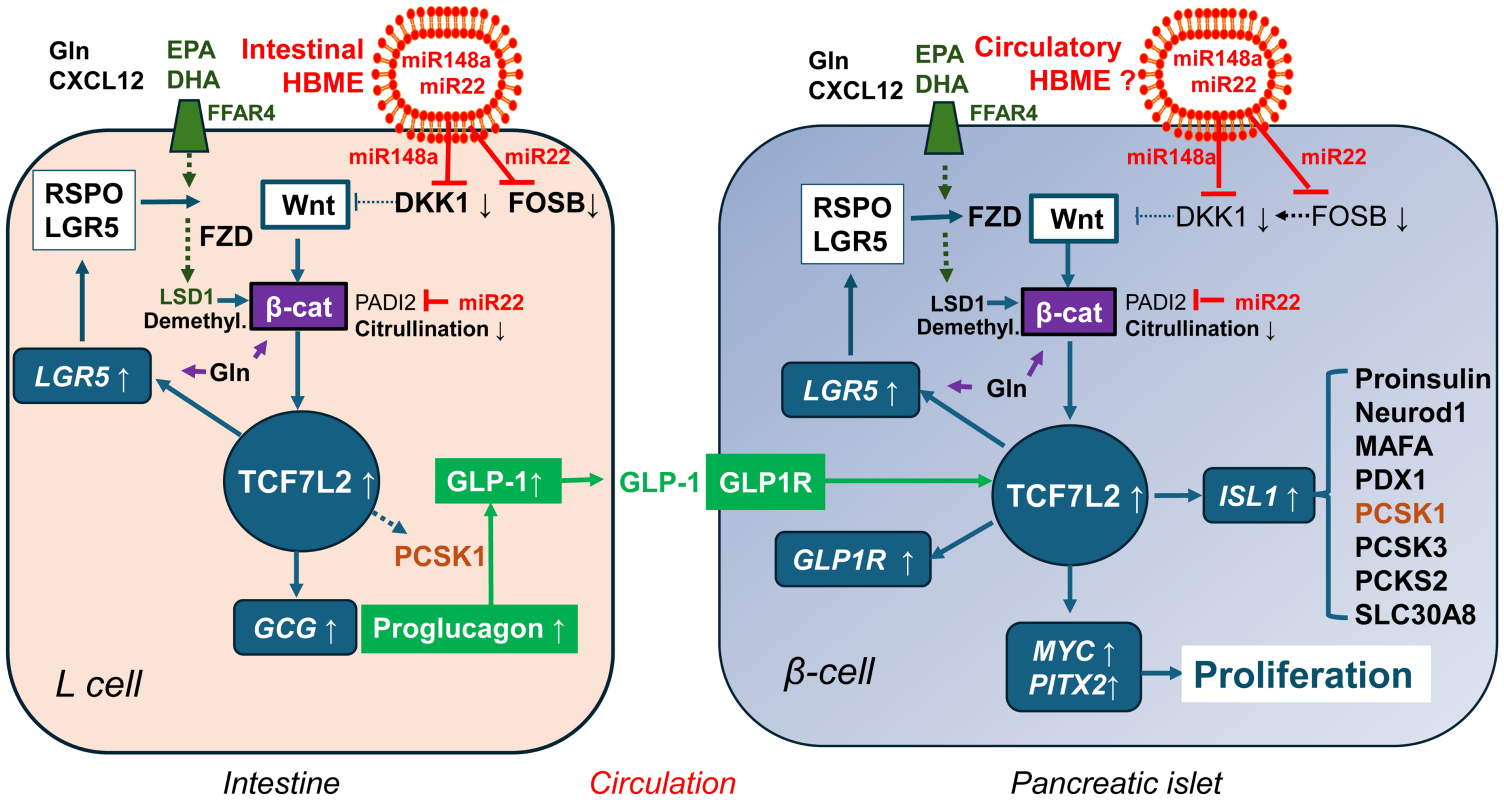
Milk-controlled Wnt crosstalk between enteroendocrine L-cells and pancreatic β-cells. Human breastmilk exosomes (HBME) are taken up by intestinal stem cells and further differentiated L-cells. The most abundant miRNAs of early lactation (miR-148a and miR-22) directly or indirectly (via suppression of *FOSB*) target the Wnt inhibitor Dickkopf 1 (DKK1) resulting in enhanced Wnt signaling. In addition, miR-22 targets peptidyl arginine deiminase 2 (PADI2). This attenuates β-catenin (β-cat) citrullination, which increases β-catenin stability. Compared to infant formula, human colostrum and human milk provide higher quantities of eicosapentaenoic acid (EPA) and docosahexaenoic acid (DHA), which via binding to free fatty acid receptor 4 (FFAR4) results in increased expressions of lysine-specific demethylase 1 (LSD1). LSD1-mediated demethylation of β-catenin enhances its nuclear stability thus augmenting Wnt signaling. In contrast to formula, human milk provides higher amounts of free glutamine (Gln), which promotes intestinal stem cell proliferation and secretion of glucagon-like peptide 1 (GLP-1). Human milk also contains GLP-1 and the chemokine CXCL12, which additionally stabilizes β-catenin. These pathways converge in activating TCF7L2 and GLP-1 generation, which interacts with TCF7L2 signaling of the β-cell. Whether or not and how long HBME reach β-cell precursors or differentiated β-cell cells is an open research question. However, systemic nutrigenomic effects of Gln and EPA/DHA may also affect Wnt-regulated β-cell development. Upregulated TCF7L2-mediated gene expression by various convergent milk factors - all deficient in formula - may promote sustained β-cell proliferation and mass expansion, explaining the potential diabetes-preventive effect of breastfeeding.

It remains an open question whether HBMEs may reach the pancreatic islet, stimulate progenitor cells or stem cells and enhance the pool of β-cells during a critical postnatal window that is important for postnatal β-cell proliferation and mass expansion. Intestinal permeability is highest directly during the first week after birth (132). In 3-6-day-old human neonates, intestinal permeability decreases in both term and preterm neonates (132, 133). In the first postnatal month, intestinal permeability of preterm infants significantly decreases for infants receiving BF versus FF in a dose-related manner (134). Thus, HBMEs, which represent the smallest EVs of breastmilk exhibiting a diameter of 50–265 nm (135, 136), may have a higher chance of transmission into the blood stream during the first week postpartum. Bovine milk exosomes are taken up by endocytosis in intestinal cells (137, 138) and vascular endothelial cells (139) allowing the conclusion that milk exosomes are bioavailable (140) and play a systemic role in epigenetic (141) and metabolic regulation (142). Colostrum-derived exosomes compared to exosomes of mature milk, may have the highest ability for direct interaction with β-cell progenitors due to the increased postnatal permeability of the gut immediately after birth. However, HBMEs of mature milk via upregulation of EEC-mediated incretin signaling may maintain an indirect communication with β-cells compared to HBME-free formula during later stages of lactation (103) (Figure 5).

## 7 The impact of exosomes on Wnt signaling

The discovery that milk exosomes influence Wnt signaling in recipient stem cells (99–101, 131) raises the question about the involvement of Wnt proteins in exosome generation, cargo composition, secretion, and transport to recipient cells. Wnt signaling plays crucial roles in development with hydrophobic Wnt proteins acting as morphogens that regulate patterning and cell differentiation over a distance through exosome-mediated transfer (143–145). Gross et al. (143) demonstrated a conserved role of exosome transport in Wnt protein distribution, showing that Wnts are secreted on exosomes both during *Drosophila* development and in human cells. Exosomes carry Wnts on their surface to activate Wnt signaling in target cells, facilitatd by the cargo receptor Evi/WIs and the R-SNARE Ykt6 (143). A specific Wnt signal peptide known as the exosome binding peptide, has been identified to guide Wnt secretion to exosomes (146). Additionally, soluble Frizzled-related proteins have been found to enhance exosome-mediated Wnt re-secretion in cells (147). The significance of exosome-Wnt interactions in central nervous system development has been recently explored (148). Overall, accumulating evidence suggests that exosomes play a role in the long-distance spreading of Wnt signaling. However, the characterization of Wnt proteins and carriers in milk exosomes remains to be investigated.

## 8 Milk exosomal miRNAs promote stem/progenitor cell Wnt signaling

The most abundant miRNA in human breast milk and milk exosomes is miRNA-148a-3p (149–153). This miRNA is overexpressed in milk and HBMEs of mothers who give birth prematurely (154, 155), while levels of miRNA-148a-3p are reduced in HBMEs of mothers with obesity (156) and gestational diabetes (157). Levels of miR-148a-3p, an oxytocin-dependent miRNA (152), are also decreased in human milk after cesarean section (158). Notably, formula does not contain exosomes or significant amounts of miRNAs (159). In fact, the level of miR-148a in infant formula is lower than 1/500th of those in mature breastmilk (158). The low amounts of miRNA-148a in formula compared to breastmilk have been confirmed recently (160).

Remarkably, Sheng et al. (161) showed that the Wnt inhibitor Dickkopf 1 (*DKK1*) is a target gene of miRNA-148a-3p. The activation of Wnt signaling by miRNA-mediated targeting of *DKK1* is the recent focus of hair research for the activation of anagen hair growth (162, 163). The observed hair regeneration by colostrum-derived exosomes associated with increased Wnt signaling (131) may be related to DKK1 suppression mediated by exosomal transfer of miRNA-148a-3p. There is recent interest in the inhibition of DKK1 to propagate Wnt/β-catenin signals as an effective way to treat related diseases (164). Increased circulating levels of DKK1 have been observed in patients with T2DM and cardiovascular disease (165).

In analogy to miRNA-148a-3p, miRNA-22-3p is also abundantly expressed in colostrum (166) and milk and HBMEs of mothers with term and especially preterm delivery (138, 167, 168). Jiang et al. (169) showed that miRNA-22 mimics promote intestinal proliferation suggesting that miRNA-22-3p from milk exosomes may promote intestinal proliferation in early life. As already outlined, milk exosomes increase the proliferation of LGR5 + ISCs (98–101) and LGR5^+^ hair follicle stem cells (131). As *LGR5* is a Wnt target gene, miRNA-22 may upregulate Wnt signaling. In fact, it has recently been shown in cutaneous squamous cell carcinoma that miRNA-22 promotes stem cell traits via activating Wnt/β-catenin signaling (170). MiRNA-22 plays a critical role in hair follicle stem/progenitor cell differentiation and facilitates β-catenin stabilization through directly repressing peptidyl arginine deiminase 2 (PADI2). PADI2 catalyzes the citrullination of β-catenin, which promotes its degradation thus suppressing Wnt signaling (170, 171). MiRNA-22-3p also targets the Wnt signaling inhibitor secreted frizzled-related protein 2 (*SFRP2*) (172). In contrast, miR-22 knockout mice showed attenuated Wnt/β-catenin activity and LGR5^+^ cancer stem cell penetrance (173).

Intriguingly, miR-22-3p also relieves DKK1-mediated repression of Wnt/β-catenin signaling by targeting a FOSB-DKK1 transcriptional axis (173). FOSB, which upregulates the expression of DKK1, is a direct target of miR-22-3p. Notably, *FOSB* is also a predicted target gene of miRNA-148a-3p (174). MiRNA-148a-3p has recently been identified as one of the key miRNAs controlling epidermal and hair follicle stem/progenitor cells (175). Thus, these most abundant HBME miRNAs of early lactation, which are overexpressed in colostrum and milk for preterm infants (138, 166–168), synergize in activating Wnt signaling promoting stem cell compartments, a meaningful maternal developmental boost for the preterm baby that may also affect islet progenitor cells. In contrast to the observed Wnt promoting effects of miRNA-22-3p, Kaur et al. (176) reported that elevated hepatic expression of miRNA-22-3p silenced Tcf7 and impaired gluconeogenesis.

Of note, the levels of miRNA-125b-5p and miRNA-148a-3p in milk were higher in transition milk (measured 4–5 days postpartum) compared to mature milk (158). Freiría-Martínez et al. (177) reported that miRNA-125b-5p levels are higher in preterm colostrum compared to term colostrum. In infant formula, miRNA-125b-5p levels were lower than 1/100th compared to those in mature breastmilk (158). Remarkably, miRNA-125b-5p also activates Wnt signaling via targeting negative regulators of Wnt signaling including DKK3, ZNRF3, RNF43, and APC2 (178). Exosomal miRNA-125b-5p derived from cancer-associated fibroblasts decreased the expression of adenomatous polyposis coli (APC) and enhanced cancer cell proliferation (179).

MiRNA-21-5p is another highly abundant miRNA found in human milk (149, 180) and HBME (150, 180). Mutai et al. (181) demonstrated that miRNA-21-5p from bovine milk exosomes is bioavailable in humans. Exosomes containing miRNA-21-5p are efficiently taken up by cardiac stem cells and reduce the expression of phosphatase and tensin homolog (PTEN) (182). Importantly, the suppression of PTEN by miRNA-21-5p enhances the activity of the kinase Akt (183). Akt-mediated phosphorylation of GSK3-*β* has been shown to enhance stem/progenitor cell enrichment by activating the Wnt/β-catenin pathway (184). The Wnt inhibitor *DDK2* is also targeted by miRNA-21-5p (185, 186).

Thus, the most abundant HBME miRNAs in early lactation activate canonical Wnt/β-catenin signaling at various regulatory points, a gene-regulatory network which is absent in FF. Jacovetti et al. (187) reported that postnatal β-cell maturation is linked to changes in islet-specific miRNAs induced by nutrient shifts at weaning, suggesting miRNAs play a central role in postnatal β-cell maturation and adult functional β-cell mass determination. In rat islets, a significant decrease in miRNA-17-5p was observed at weaning, a period associated with a transition from β-cell proliferation and mass expansion to β-cell maturation (187). Weaning leads to a decrease in the transfer of exosomal milk miRNAs (188). Jaafar et al. (189) reported that the control of cellular signaling in β-cells fundamentally changes after weaning, shifting from the nutrient sensor mTORC1 to the energy sensor AMPK, which is crucial for functional β-cell maturation, mitochondrial biogenesis, and GSIS, all of which have been linked to weaning-related changes in milk miRNA signaling (129, 188). Notably, miRNA-17-5p is upregulated in moderate/very preterm colostrum compared to moderate/very preterm mature milk (177). In hepatic stellate cells, miRNA-17-5p activates Wnt/β-catenin signaling by inhibiting Wnt inhibitory factor 1 (*WIF1*) expression (190–192). Therefore, miRNA-17-5p may also promote Wnt signaling during early lactation.

## 9 Long-chain polyunsaturated *ω*-3 fatty acids promote stem cell Wnt signaling

Polyunsaturated fatty acids play an important role in early infant development (193, 194). The vast majority of infant formulas in the United States contain the long-chain polyunsaturated fatty acids (PUFAs) docosahexaenoic acid (DHA) and arachidonic acid, which were first permitted by the US Food and Drug Administration in 2001 (195). However, compared to human milk (196), infant formulas contain significantly lower levels of eicosapentaenoic acid (EPA) and DHA (197). Granot et al. (198) reported that the percentage of ω-3 fatty acids of total red blood cell phospholipid fatty acids was significantly higher in breastfed infants than in formula-fed infants (6.31 ± 2.5% compared with 2.98 ± 0.97%). DHA concentrations were also significantly higher in breastfed infants (5.1 ± 1.2%) compared with formula-fed infants (2.2 ± 0.9%, *p* < 0.001). Of note, EPA and DHA bind to free fatty acid receptor 4 (FFAR4, also known as GPR120) (199, 200), which upregulates the expression of miRNA-30b-5p promoting brown thermogenesis (201) and stimulates intestinal secretion of GLP-1 (202). In a murine model of dextran sulfate sodium (DSS) induced colitis, EPA increased the numbers of proliferative cells, absorptive cells, goblet cells, and GLP-1-producing EECs (203). EPA-mediated upregulation of FFAR4 increased the expression of lysine-specific demethylase 1 (LSD1), which facilitated ISC proliferation and differentiation in organoids (203). Remarkably, EPA administration activated the Wnt signaling pathway downstream of LSD1 in ISCs, while inhibiting Wnt signaling abolished the beneficial effects of EPA (203). LSD1 has recently been identified to control a nuclear checkpoint in Wnt/β-catenin signaling regulating muscle stem cell self-renewal (204). Demethylation of β-catenin by LSD1 prevents its degradation, thereby maintaining its nuclear levels (204). LSD1 also demethylates H3K4me1/2 and H3K9me1/2 at target loci in a context-dependent manner, influencing the stem cell state, including the regulatory circuitry underlying self-renewal and pluripotency (205). Lei et al. (206) showed that LSD1 promotes β-catenin activation by inhibiting the expression of several suppressors of β-catenin signaling, especially prickle planar cell polarity protein 1 (PRICKLE1) and APC in LGR5^+^ liver cancer-initiating cells, by directly regulating the levels of mono- and di-methylation of histone H3 lysine-4 at the promoters of these genes. Prickle-1 negatively regulates the Wnt/β-catenin pathway by promoting Dishevelled ubiquitination/degradation (207). Notably, *PRICKLE1* is a predicted conserved targeted gene of miRNA-30b-5p (208), which is upregulated by EPA-mediated activation of FFAR4 (199).

LSD1-associated activation of the β-catenin signaling is essential for maintaining the activity of LGR5^+^ liver cancer initiating cells (206). Vinckier et al. (209) verified LSD1 expression in pancreatic progenitor cells and differentiated endocrine cells in human fetal and adult tissue. They also showed in mice that LSD1 is required for endocrine cell formation during a short window in early pancreatic development. LSD1 also plays a key role in the regulation of pancreatic islet progenitor cells (209), while LSD1 inhibition promoted the differentiation of insulin-producing cells from human ESCs and human induced pluripotent stem cells (210, 211). A novel demethylase-independent role of LSD1 in regulating gene expression and cell fate transition of ESCs has recently been reported (212).

## 10 Glutamine promotes stem cell Wnt signaling and β-cell mass expansion

Breastfed infants receive ample free amino acids (FAA) (213–215), primarily glutamic acid (Glu) and glutamine (Gln), compared to formula-fed babies (213). In human milk, Glu, Gln, and taurine are the most abundant amino acids, making up about 50% of total FAAs. However, in analyzed formulas, the total FAA fraction is only 10% or even less than in human milk, and the combined amount of Glu and Gln in all formulas is much lower than in human milk (213). A study by Agostoni et al. (216) found that breastfed infants receive increasing amounts of Gln and Glu as lactation progresses. Glu, the prevalent FAA, and Gln increase approximately 2.5 and 20 times, respectively as lactation continues, making up more than 50% of total FAAs by 3 months (216). FAAs play a role in neonatal immune development and contribute to the unique protective effects of BF (217). Chuang et al. (218) confirmed that the average concentration of total FAAs in human milk (8,139 μmol/L for preterm human milk; 3,462 μmol/L for full term human milk) is significantly higher than in any infant formulas (powdered term formula TF-A, 720 μmol/L; TF-B, 697 μmol/L; and preterm formula PTF-A, 820 μmol/L; PTF-B, 789 μmol/L) (*p* < 0.01). The concentration of FAAs is highest in human colostrum and decreases as milk transitions to the mature milk stage (218).

Accumulated evidence points to an important role for Gln in the expansion of ISCs and intestinal cell proliferation (219–222). Tian et al. (223) recently observed that Gln upregulates Wnt signaling in ISCs by studying early weaning mice and intestinal organoids. They found that Gln ameliorated early weaning-induced epithelial atrophy and enhanced ISC-mediated epithelial regeneration. Gln accelerates ISC-mediated intestinal epithelial development by increasing Wnt signaling. When Gln was added to enteroids, mRNA expression levels of Wnt-responsive genes like β-catenin, c-Myc, Cd44, Axin2, LGR5, and LGR6 increased. Inhibition of Wnt signaling negated the effects of Gln on ISCs (223). Fang et al. (224) confirmed that Gln promotes porcine intestinal epithelial cell proliferation through activation of the Wnt/β-catenin pathway. Gln increased cytosolic and nuclear β-catenin protein expression. An LF3 assay (a β-catenin/TCF4 interaction inhibitor) and β-catenin knockdown blocked Gln-mediated promotion of Wnt/β-catenin signaling and cell proliferation. Additionally, the inhibition of TCF4 expression suppressed Gln-induced cell proliferation. Gln-mediated activation of Wnt signaling may not only affect ISCs and IECs but also activate intestinal L-cells. In fact, Chen et al. (225) observed that Gln enhanced the expression of chromogranin A, a differentiation marker of EECs, in ISCs and organoids. Furthermore, Gln stimulates the secretion of GLP-1 in L-cells (226, 227). Oral Gln has been shown to increase circulating levels of GLP-1, glucagon, and insulin in lean, obese, and T2DM subjects (228).

Modi et al. (229) demonstrated that Gln stimulates the biosynthesis and secretion of insulin-like growth factor 2 (IGF2), an autocrine regulator of β-cell mass and function. Exposure of insulinomas or β-cells to Gln induced Akt phosphorylation (229), which via inhibitory phosphorylation of GSK-3*β* enhances Wnt signaling (230). Gln also modulates protein translation through mTORC1 in β-cells (231, 232). Furthermore, AAs play an important role in the proinsulin pool. β-cells avidly consume extracellular Gln, serine, and cysteine (233). Thus, insufficient postnatal supply of Gln by FF adversely affects Wnt signaling of the entero-islet axis (L-cells and β-cells), which compromises postnatal Gln and GLP-1-stimulated Wnt-driven β-cell proliferation and mass expansion (20).

## 11 CXCL12 and TCF7L2-mediated β-cell proliferation

The CXC chemokine CXCL12, also known as stromal cell-derived factor-1 (SDF-1), is a crucial signaling component in both physiological and pathological processes (234). CXCL12 functions by interacting with the CXC chemokine receptor 4 (CXCR4), which leads to the activation of Akt (235). Akt inhibits GSK-3*β* and phosphorylation of β-catenin by GSK3, thus preventing degradation of β-catenin, and resulting in stabilization of β-catenin, which accumulates in the cytoplasm, enters the nucleus, and associates with TCF7L2. Therefore, CXCL12 promotes the survival of pancreatic β-cells by stabilizing β-catenin and activating TCF7L2 (235). Transgenic mice expressing CXCL12 in their β-cells are protected against streptozotocin-induced diabetes through the activation of the pro-survival protein kinase Akt and downstream pro-survival, anti-apoptotic signaling pathways (236). A study on CXCL12-activated Wnt signaling in isolated islets and INS-1 cells using a β-catenin/TCF-activated reporter gene assay showed enhanced Wnt signaling through the Gαi/o-PI3K-Akt axis, suppression of GSK3β, and stabilization of β-catenin (235). Additionally, CXCL12 signaling in INS-1 β-cells stimulates the accumulation of β-catenin mRNA, likely due to enhanced transcription of the β-catenin gene (235). Kayali et al. (237) confirmed that the CXCL12/CXCR4 axis is necessary for the proliferation and maturation of human fetal pancreatic endocrine progenitor cells. The signaling mechanisms of CXCL12 could be utilized to modulate β-cell autoimmunity, protect and preserve functional β-cell mass, and for cell replacement therapy in T1DM (238). The importance of the CXCL12/CXCR4 axis for diabetes therapy has been extensively reviewed (239).

Remarkably, activation of the CXCL12/CXCR4 axis induces intra-islet GLP-1 production and enhances β-cell survival (240). CXCL12 induces the expression of prohormone convertase 1/3 and subsequently leads to the production of GLP-1 in *α*-cells. The combination of GLP-1 and CXCL12 synergistically enhances both the growth and longevity of INS-1 β-cells (240). Interestingly, there are similarities in the processing of proglucagon to GLP-1 in islet α-cells and enteroendocrine L-cells (241). It is therefore plausible that CXCL12 may also stimulate the processing of proglucagon to GLP-1 in enteroendocrine L-cells. Intriguingly, CXCL12 has been identified as a component of human colostrum and breastmilk (242, 243). Additonally, He et al. (244) reported that human colostrum oligosaccharides enhance the expression of CXCL12 in the immature human intestine.

Exosomes have the potential to carry CXCL12 (245). Studies have shown that exosomal miRNAs can enhance CXCL12 expression (246, 247). Exosomal CXCL12 has been further found to upregulate the expression of miRNA-125b (248). Importantly, the reciprocal positive feedback loop between CXCR4 and miRNA-125b further activates the Wnt/β-catenin signaling pathway by targeting the *APC* gene (248). However, to date, no study has investigated the potential relationship between milk exosomes and the transfer of exosomal CXCL12, or the exosomal miRNA-mediated expression of CXCL12 and CXCL12-mediated miRNA expression. Furthermore, there is a lack of data on the CXCL12 content of infant formulas compared to human milk, and no studies have characterized the impact of formula on CXCL12 and entero-islet signaling.

## 12 Human milk’s Wnt signaling network for developing islet and adipose tissue stem cells

Collected translational evidence supports the view that human milk provides various components including exosomes, miRNAs, free amino acids, *ω*-3 fatty acids and chemokines that may all converge in promoting developmental Wnt signaling. Among the various tissue sources known to secrete exosomes that traffic to pancreatic islets (249), lactating mammary gland-derived HBME may especially serve as a further physiological route of exosome signaling supporting Wnt-dependent β-cell progenitors during the early postnatal lactation period. Future studies with clonal pancreas organoids may allow deeper insights into the potential effects of HBMEs on β-cell precursors. Unlimited *in vitro* expansion of adult bi-potent pancreas progenitors through the LGR5/R-spondin axis with differentiation into endocrine cells has already been reported (250). Recently, Kim et al. (251) observed significant differences in exosomal miRNA patterns in serum and urine in preterm infants fed with either breast milk or infant formula.

In analogy to adipocyte-derived stem cells (62, 63, 65), increased mTORC1/S6K1 signaling in β-cell progenitors induced by FF (58, 59) may suppress Wnt gene expression by overstimulating nuclear activity of EZH2 (252) and reducing FZD availabily (64). A sophisticated molecular interplay of the various milk components maintains appropriate communication for Wnt stem cell signaling in the recipient tissues of the infant. In addtion, human milk transfers stem cells with exciting therapeutic potential (253–257) and maintains an intrinsic epigenetic cross talk that promotes the infant’s stem cell niches. The ability of milk exosomes to stimulate LGR5^+^ stem cell growth suggests their potential use in tissue regeneration and possibly in diabetes treatment (258, 259). However, prolonged activation of Wnt/LGR5 by milk exosomes beyond the postnatal lactation period may increase the risk of Wnt-driven cancers (98, 260–264). Activation of Wnt signaling by BF may boost precursor β-cell growth and support postnatal transformation of ductal or acinar progenitors into insulin-producing β-cells. Understanding the timing and location of postnatal β-cell development in relation to the postnatal nutritional environment could help slow the diabetes epidemic. It is agreed with Lönnerdal’s opinion (265) that formula composition does not mimic breastmilk changes over time. In contrast to “static” FF, human milk’s complex and changing composition throughout lactation provides the necessary calibration and magnitude of Wnt signaling crucial for postnatal state of β-cell development. This may explain the molecular basis for BF’s diabetes-preventive effects. Table 1 summarizes selected key components of human milk that support enteroendocrine stem cell Wnt signaling and are lacking in infant formula.

**Table 1:** Potential diabetes-preventive components in human milk that activate stem cell Wnt signaling but are deficient or insufficient in infant formula.

Human milk and milk components	Potential impact on the enteroendocrine-islet axis and Wnt-driven β-cell expansion	Reference
Human milk	Human milk-supplemented organoids compared to formula induce higher differentiation of enteroendocrine cells and increase Wnt signaling.	(103)
Milk exosomes	Milk exosomes lead to an increase in the proliferation of LRG5^+^ intestinal stem cells, the number of ileum crypts, and the expression of LGR5 in intestinal stem cells.	(99, 101)
miRNA-148a-3p	This miRNA increases Wnt signaling by targeting the Wnt inhibitor Dickkopf 1 (DKK1).	(161)
miRNA-22-3p	It promotes proliferation of human intestinal epithelial cells and increases Wnt signaling by targeting peptidyl arginine deiminase 2 (PADI2), which catalyzes the citrullination and subsequent degradation of β-catenin. It further increases Wnt signaling by targeting the Wnt inhibitor secreted frizzled-related protein 2 (*SFRP2*) and relieves DKK1-mediated repression of Wnt signaling by targeting a FOSB-DDK1 transcriptional axis. *FOSB* is a direct target of miRNA-22-3p and miRNA-148a-3p	(169–173)
miRNA-125b-5p	This miRNA increases Wnt signaling by targeting negative regulators of Wnt signaling including DKK3, ZNRF3, RNF43, and APC.	(178, 179)
miRNA-21-5p	This miRNA leads to increased Wnt signaling by suppressing PTEN, enhancing Akt-mediated GSK3-β suppression, and suppressing the Wnt inhibitor DDK2.	(183–186)
miRNA-17-5p	It triggers increased Wnt/β-catenin by targeting Wnt inhibitory factor 1 (WIF1).	(190–192)
miRNA-30b-5p	It induces increased Wnt signaling by targeting prickle planar cell polarity protein 1 (PRICKLE1) and promoting Dishevelled ubiquitination and degradation.	(207, 208)
Eicosapentaenoic acid	This compound increases secretion of GLP-1 and Wnt signaling by enhancing the expression of lysine-specific demethylase 1 (LSD1) demethylating β-catenin, thereby preventing its degradation. It further increases intestinal stem cell proliferation and differentiation. LSD1 promotes Wnt signaling by inhibiting the Wnt inhibitors prickle planar cell polarity protein 1 (*PRICKLE1*) and APC regulator of Wnt signaling pathway (*APC*) by directly regulating the levels of mono- and di-methylation of histone H3 lysine-4 at the promoters of these genes.	(202, 204, 206)
Glutamine	This amino acid upregulates Wnt signaling and LGR5 expression in intestinal stem cells. It was shown to increase Wnt signaling and the proliferation of porcine intestinal epithelial cells. It further enhances the expression of L-cell marker chromogranin A and the secretion of GLP-1 in L-cells. Additionally, it triggers biosynthesis and secretion of insulin-like growth factor 2 (IGF2), an autocrine regulator of β-cell mass and function. Furthermore, it provokes Akt phosphorylation, which enhances Wnt signaling by inhibitory phosphorylation of GSK-3β. It increases protein translation through mTORC1 in β-cells.	(223–227, 229–231)
CXCL12	This chemokine increases Wnt signaling by CXCR4-mediated activation of Akt, resulting in Akt-mediated inhibition of GSK3.	(235, 236)

BF has been identified as a critical preventive postnatal condition for both T2DM and obesity. BF not only calibrates the correct magnitude of postnatal Wnt/β-catenin/TCF7L2 signaling in the pancreatic islet stem cell niches but also in adipose tissue stem cell (ASC) niches. Compromised Wnt signaling by FF may explain exaggerated ASC commitment paving the way to obesity (252). The canonical Wnt signaling is regarded as a significant endogenous inhibitor of adipogenesis (252, 266). Intriguingly, Boyle et al. (267) showed that umbilical cord MSCs from infants born to obese mothers (Ob-MSCs) compared to MSCs of infants of mothers with normal weight (NW-MSCs) exhibit greater potential for adipogenesis. Ob-MSCs showed altered GSK-3β/β-catenin signaling in MSCs due to increased β-catenin degradation, resulting in 10% lower β-catenin levels compared to NW-MSCs. In fact, the ultimate Wnt pathway effector TCF7L2 has recently been identified as a critical regulator of adipocyte development (268). Inactivation of TCF7L2 protein in mature adipocytes *in vivo* leads to whole-body glucose intolerance, hepatic insulin resistance, increased subcutaneous adipose tissue mass and adipocyte hypertrophy (268). It is important to note that TCF7L2 mRNA is upregulated in islets in diabetes, but TCF7L2 protein levels are downregulated (269).

Weight loss following gastric bypass surgery resulted in the differential expression of TCF7L2 mRNA isoforms in subcutaneous fat (270). The expression of the short mRNA variant, which lacks exons 12, 13, and 13a (Ex12-, 13-, 13a-) decreased after weight loss in subcutaneous fat and liver but is more prevalent in the subcutaneous fat of individuals with T2DM (269). Overexpression of the short (Ex12, 13-, 13a-) mRNA variant induced β-cell apoptosis, while a variant containing Ex12, but not Ex13 or Ex13a, had a protective effect on β-cell survival (270). It is currently unknown whether FF compared to BF shows differences in TCF7L2 mRNA splicing.

## 13 Conclusion

**Figure 6:**
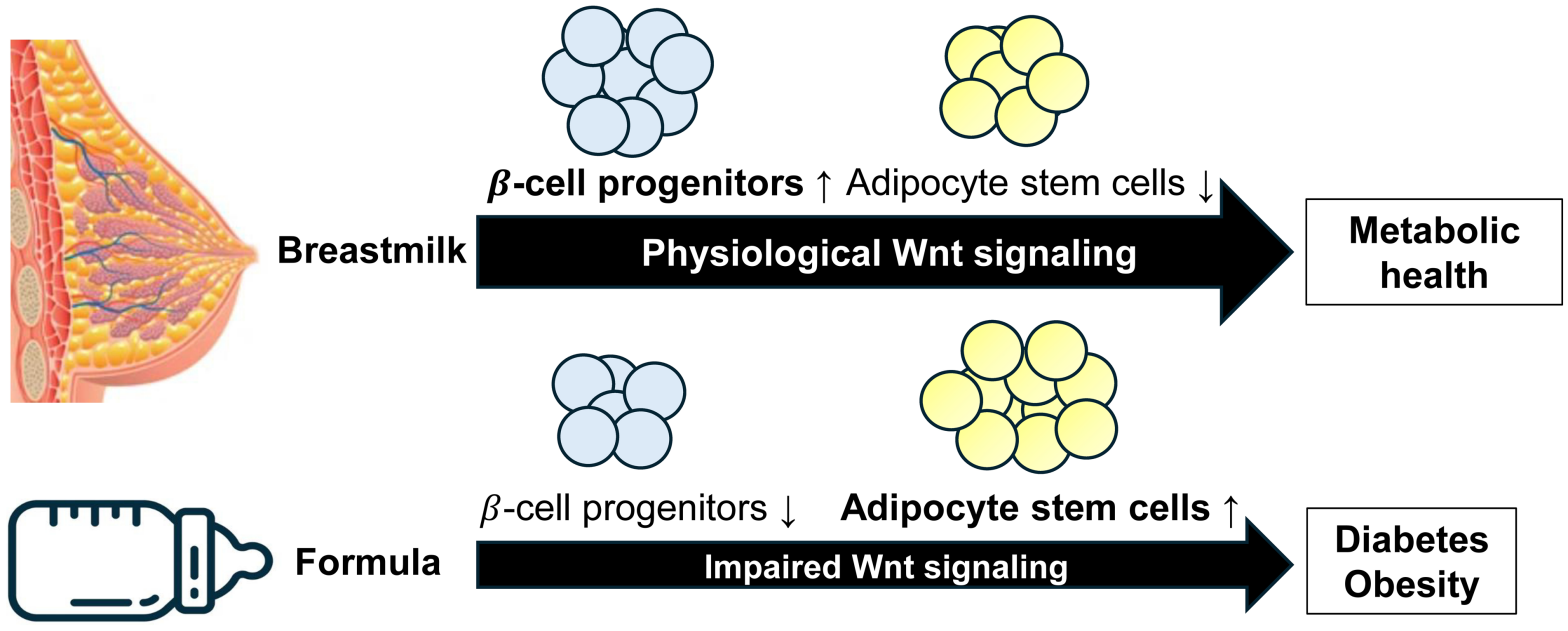
Model illustrating deviated Wnt stem signaling by artificial formula feeding versus physiological breastfeeding. Breastfeeding maintains the appropriate Wnt signaling magnitude and kinetics required for the regulated development of β-cell progenitor cells and adipocyte stem cells. In contrast, formula feeding impairs the amplitude of Wnt signaling diminishing the pool of β-cell progenitor cells and reducing the postnatal pool of β-cells. Furthermore, reduced Wnt signaling enhances the commitment of adipocyte stem cells to adipogenesis. Formula feeding is thus a priming factor for both diabetes and obesity.

Human milk transmits signals from the lactation genome to the infant, carrying out a complex postnatal program to maintain the appropriate level of Wnt signaling for stem cells in the intestine, pancreatic islets, adipose tissue and other organs. Based on translational evidence, we can deduce that BF leads to higher Wnt signaling compared to FF, which promotes the expansion of β-cell mass, explaining the preventive effect of BF on diabetes. However, BF suppresses ASC commitment, which explains its preventive effect on obesity (Figure 6). Wang et al. (65) confirmed that increased Wnt signaling encourages the differentiation of ASCs into islet β-cells. Therefore, milk-regulated Wnt signaling plays a significant role in crucial stem cell fate determinations. We propose that infants born to obese mothers with impaired fetal MSC Wnt signaling are at a significant risk of developing diabetes if they are formula-fed, perpetuating perinatal abnormalities from the normal Wnt signaling pathway.

Accumulated evidence disproves the historical pediatric understanding of milk as “just food,” which was an oversimplified and misleading perception that enabled the implementation of artificial infant FF (271). The defining trait of mammals is their mammary glands, which are used to raise their offspring. These glands not only provide a food source to nourish the infant but simultaneously program the young. “Breastfeeding” is thus a misleading and restricted term as the mammary gland executes the program of lactation facilitating feeding and programming the offspring. The natural maternal transmission of breastmilk is the unchallenged gold standard for physiological postnatal infant development, whereas artificial formula perturbs Wnt-controlled stem cell homeostasis paving the way for diabetes and obesity.

## 14 Future perspectives

For a deeper understanding of the epigenetic and nutrigenomic differences of Wnt signaling between BF and FF and its regulatory impact on pancreatic stem cell progenitor cells, future research should address the following questions:

1. Do deficiencies in the amounts and kinetics of milk miRNAs, Gln, EPA, and DHA during a vulnerable postnatal period of Wnt-driven β-cell development in infants fed formula prime the risk of diabetes later in life?
2. Does FF-induced overactivation of Akt via GSK-3β inhibition partially compensate for the deficient Wnt regulating mechanisms provided by BF?
3. Do milk exosomes enter the systemic circulation and reach the islets during the postnatal β-cell mass expansion and is Wnt signaling involved in the production and secretion of milk exomes?
4. Do milk exosomes transfer Wnt components and R-spondins or their corresponding mRNAs to recipient cells and does the addition of milk exosomes enhance the number of LGR5^+^ progenitors in pancreatic organoid cultures?
5. Do milk-derived stem cells and milk exosomes cooperate synergistically in postnatal islet progenitor cell development and does donor milk processing with a reduction in milk exosomes impair milk’s capacity for Wnt signaling?
6. Do milk exosomes carry activating components of the Wnt signaling pathway?
7. Is milk the optimized conditional medium for the growth and survival of milk stem cells and do FF and BF differ in CXCL12/CXCR4 signaling of the entero-islet axis?
8. Are infants carrying the TCF7L2 variant rs7903146 risk allele associated with increased tisk to develop T2DM by FF, and does FF modify the generation of TCF7L2 splice variants?

We hope that our review will inspire future research in these areas.

## Glossary

AA: amino acid
ADSC: adipose-derived stem cell
AKT: serine/threonine kinase
AMPK: AMP-activated protein kinase
APC: adenomatosis polyposis coli
BF: breastfeeding
BCAA: branched-chain amino acids
cAMP: cyclic adenosine monophosphate
CK1: casein kinase 1
COUP-TF2: chicken ovalbumin upstream promoter transcription factor 2
CTNNB1: catenin beta 1 gene
CXCL12: chemokine, CXC motif, ligand 12 (SDF-1)
CXCR4: chemokine receptor 4
DHA: docosahexaenoic acid
DKK1: Dickkopf 1
DKK2: Dickkopf 2
DKK3: Dickkopf 3
EBP50: ERM-binding phosphoprotein 50
EPA: eicospentaenoic acid
ESC: embryonic stem cell
EV: extracellular vesicle
EVI: evenness interrupted, Drosophila, homolog of
Exd4: exendin-4
EZH2: enhancer of zeste homolog 2
FAA: free amino acid
FF: formula feeding
FFAR4: free fatty acid receptor 4 (GPR120)
FOSB: FOSB protooncogene, AP1 transcription factor subunit FOSB
FZD: Frizzled receptor (FZD)
GCG: proglucagon gene
GIP: glucose-dependent insulinotropic polypeptide
GIPR: GIP receptor
GLP-1: glucagon-like peptide 1
GLP1R: GLP-1 receptor
GLUT2: glucose transporter type 2
GSIS: glucose-stimulated insulin secretion
GSK3: glycogen synthase kinase 3
H3K27me3: histone 3 trimethylated at lysine 27
HMDE: human milk derived exosomes
hucMSC: human umbilical cord mesenchymal stem cell
IEC: intestinal epithelial cell
IGF-1: insulin-like growth factor 1
IGF1R: IGF-1 receptor
IGF-2: insulin-like growth factor 2
IPC: insulin-producing cell
IR: insulin receptor
ISC: intestinal stem cell
ISL1: ISL LIM homeobox 1
LAT: L-type amino acid transporter
LGR5: leucine-rich repeat-containing G-protein-coupled receptor 5
LRP5/6: low-density lipoprotein receptor-related protein 5/6
LSD1: lysine-specific demethylase 1
MAFA: MAF bZIP transcription factor A
miRNA: micro-ribonucleic acid
MSC: mesenchymal stem cell
mTORC1: mechanistic target of rapamycin complex 1
NeuroD1: neurogenic differentiation 1
NHERF1: Na/H exchanger regulatory factor 1
NKD1: NKD inhibitor of Wnt signaling pathway 1
NKX6.1: NK6 homeobox 1
NR2F2: nuclear receptor subfamily 2, group F, member 2 (COUP-TF2)
NOTUM: NOTUM, palmitoleoyl-protein carboxylesterase
PADI2: peptidyl arginine deiminase 2
PCSK1: proprotein convertase, subtilisin/kexin-type 1
PCSK3: proprotein convertase, subtilisin/kexin-type 3
PDX1: pancreas/duodenum homeobox protein 1
PI3K: phosphatidylinositol 3-kinase
PITX2: paired-like homeodomain transcription factor 2
PKA: protein kinase A
PRICKLE1: prickle planar cell polarity protein 1
PRLR: prolactin receptor
PTEN: phosphatase and tensin homolog
PUFA: polyunsaturated fatty acid
RNF43: ring finger protein 43
RSPO: R-spondin
SDF-1: stromal cell-derived factor-1 (CXCL12)
SFRP2: secreted frizzled-related protein 2
S6K1: S6 kinase 1
SOX9: SRY-box 9
STAT5: signal transducer and activator of transcription 5
TCF3: transcription factor 3
TCF7: transcription factor 7
TCF7L2: transcription factor 7-like 2
T1DM: type 1 diabetes mellitus
T2DM: type 2 diabetes mellitus
WIF1: Wnt inhibitory factor 1
WNT: wingless
Ykt6: Ykt6 SNARE homolog
ZNRF3: zinc finger and ring finger protein 3
